# Normalized hand-behind-back for the measurement of shoulder internal rotation

**DOI:** 10.1016/j.jseint.2021.12.001

**Published:** 2022-01-10

**Authors:** Masahiro Mitsukane, Karen Suzuki, Ryusei Tabe, Fukuhiro Hasumi, Daiki Fukushima

**Affiliations:** Department of Rehabilitation Medicine, Shonan University of Medical Sciences, Yokohama, Kanagawa, Japan

**Keywords:** Glenohumeral joint, Internal rotation, Range of motion, Measurement, Hand behind back, Reliability

## Abstract

**Background:**

The hand-behind-back (HBB) is a method for measuring the range of shoulder internal rotation; here, the highest vertebral level reached by the thumb is recorded. Alternatively, other specific landmarks may be used to measure its distance with the thumb. When the records of distance are adopted, it becomes difficult to compare individuals of different physiques, that is, comparing adults and children. In this study, we proposed a modified HBB method that attempts to normalize body size disparity and examined its reliability.

**Methods:**

Three raters measured the modified HBB in 60 healthy subjects. A test-retest design was used, wherein each rater measured one trial, for a total of three trials each subject. The subject's thumb was actively and passively ascended along the spinal column as high as possible; subsequently, the distance between the C7 spinous process and tip of the thumb (C7-thumb) was measured with a tape. The HBB ratio (HBBR) was used as the parameter of shoulder internal rotation. It was defined as the ratio between the C7-thumb and the distance between the C7 spinous process and midpoint of the line connecting the posterior superior iliac spines (C7-posterior superior iliac spine).

**Results:**

Intraclass correlation coefficients (model 2.1) ranged from 0.73 to 0.89, indicating that the reliability of the active and passive HBBR had moderate or good and good reliability, respectively. Bland-Altman analysis revealed that the values of minimal detectable changes were 0.053 and 0.036 for the active and passive measurements, respectively.

**Conclusion:**

The proposed method was confirmed to have sufficient reliability for clinical use. The HBBR may be used as a parameter of the shoulder internal rotation, which enables the comparison between individuals of different physiques.

When measuring the range of shoulder internal rotation, movement of the forearm serves as the movable axis and is hindered by the subject's abdomen. Therefore, it is often performed with the arm 90° abducted position or using the hand-behind-back (HBB) method without the universal goniometer.^22^ The HBB method is also named the “vertebral method”; here, the thumb on the examined side is placed on the subject's back and is actively or passively ascended along the spinal column. The highest vertebral level the thumb can reach is recorded. This method comprises the movement of the scapula and scapulohumeral, elbow, and wrist joints; therefore, previous studies have questioned the validity of the HBB method in measuring the true range of shoulder internal rotation.^9^^,^^14^^,^^17^^,^^19^^,^^26^ Despite the satisfactory intraobserver reliability, the HBB method has poor interobserver reliability^7^; in addition, constraints may be observed because of categorical data.^25^ Nevertheless, the HBB method remains as an important measure of shoulder function as it has been related to personal functions, such as toileting and dressing, and the detection of motion loss, which is an early sign of pathology. Currently, the HBB method is endorsed by The American Academy of Orthopaedic Surgeons^1^ and American Shoulder and Elbow Surgeons^21^ as a standard for assessing shoulder internal rotation.

One factor that may affect the reliability of the HBB method is the difficulty in palpating bony landmarks. In 2002, Edward et al^7^ stated that instead of using the vertebral level, easier landmarks, such as the iliac crest, may improve the reliability of the HBB method. Correspondingly, Han et al^10^ proposed a modified HBB method that measures the distance between the tip of the thumb and the spinous process of C7 to estimate the vertebral level using a conversion formula, which had high accuracy. However, the reproducibility of the measurement was not examined. In addition, their subjects comprised adults aged >18 years; therefore, the applicability of the equation to individuals of different physiques, including children, remains unclear. On the other hand, Sharma et al^24^ proved the reliability of measuring the distance between the posterior inferior iliac spine and radial styloid as landmarks for the HBB method. However, their manner did not include any procedure of normalization in statistics, which considered the disparity in body size. In this study, we propose a modified HBB method that attempts to normalize the body size disparity, that is, the trunk length difference related to standing or sitting height. This would allow the collection of continuous variables as the parameter of shoulder internal rotation and allow comparison among individuals of different ages. In addition, the interobserver reliability of this method was examined.

## Materials and methods

### Participants

This study included healthy participants; participants with a history of illness or trauma that may cause muscular weakness were excluded.

Written informed consent was obtained from all the subjects. Ethical approval for the study was granted by the research ethics committees of SHONAN University (approval No. 21-011).

### Experimental design

Test-retest design was used, wherein each subject underwent three trials; three raters evaluated one trial each. The second trial was performed immediately after the first trial, on the same day. The third trial was performed within 7 days after the first and second trials. The order of the rater was randomized for each subject. One side of the shoulder selected randomly was measured throughout three trials.

Two occupational therapy students (raters A and B) and one occupational therapist (rater C) with >10 years of clinical experience served as the raters. Raters were not permitted to observe the other raters, and they had no access to the results. Before the start of the experiment, all raters trained for approximately 1 hour to master the measurement skill.

### Measurement

Subjects were seated straight on a chair without backrest. After positioning, the start time of measurement was recorded. The C7 spinous process and both posterior superior iliac spines (PSISs) were identified by palpation. Subsequently, the shortest distance between the spinal level of the PSIS (midpoint of the line connecting both PSISs) and the C7 spinous process (C7-PSIS) was measured with a tape. The same measurement was performed even in patients with gaps between the body surface and tape due to lumbar lordosis.

Subsequent measurement followed the manner of the HBB method. First, the subjects actively ascended the thumb along the spinal column as high as possible. Then, the distance between the C7 spinous process and the tip of the thumb (C7-thumb) was measured with a tape. Second, the same procedure was performed passively by the rater. The highest point was recorded as per the subjective feeling of the rater and the subject's pain within a tolerable level. When the mark of the thumb was located cephalad to the C7 spinous process, the value was recorded as the minus distance.

**Figure 1:**
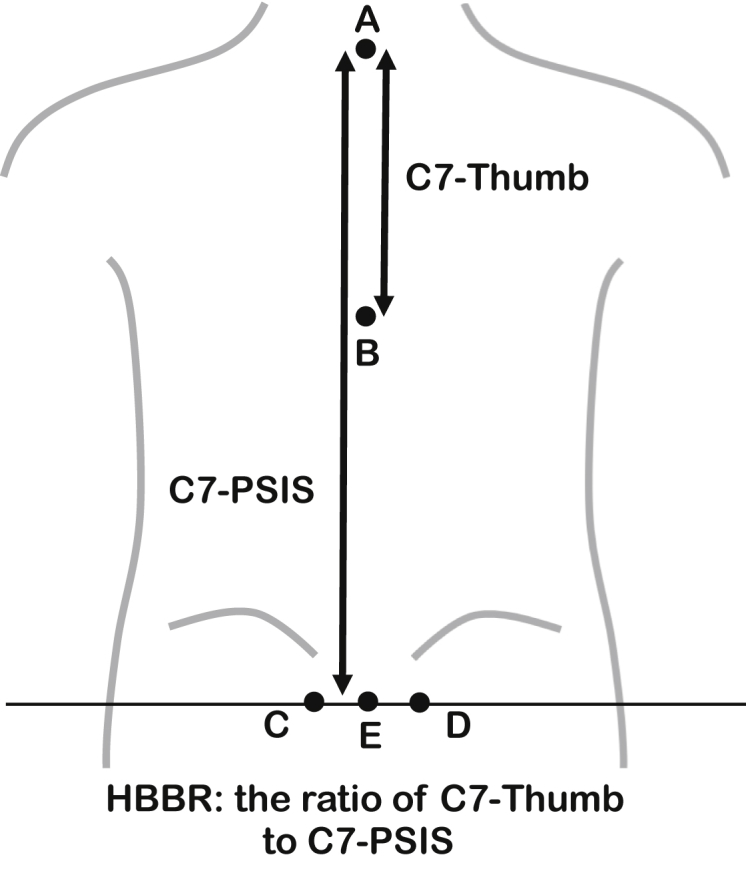
Reference line and distance measurements. A: Palpated C7 spinous process. B: Mark of the thumb. C and D: Palpated posterior superior iliac spines. E: Midpoint of the line C-D. C7-thumb: The distance between A and B. C7-PSIS: The distance between A and E. The HBBR is the ratio of C7-thumb to C7-PSIS. C7-thumb was measured both for the active and the passive motion so that the HBBR was calculated in two conditions. *PSIS*, posterior superior iliac spine; *HBBR*, hand-behind-back ratio.

The minimum unit of measurement was 1 cm. After the measurement was completed, the ratio of the C7-thumb to the C7-PSIS was calculated both for the active and passive motion. This was named as the HBB ratio (HBBR) and was defined as a parameter of the range of shoulder internal rotation (Fig. 1).

### Statistical analyses

One-way repeated measures analysis of variance (ANOVA) was performed to compare the measured values among the three trials. The Bonferroni correction was used for the post hoc analysis.

Intraclass correlation coefficients (ICCs_2,1_) and their 95% confidence intervals were determined to verify the relative inter-rater reliability: ICC <0.50 was poor; ICC 0.50-0.75 was moderate, and ICC >0.75 was good.^20^

The Bland-Altman analysis^4^ was used to account for the absolute reliability between raters. Calculations included 95% confidence intervals for the mean difference of the two paired measures, regression between the difference and mean of the two paired measures, standard error of measurement (SEM), 95% confidence interval of minimal detectable change (MDC_95_), and 95% limits of agreement (95% LOAs). The SEM was calculated using the equation SEM = SD_d_ × √1-γ ^6^^,^^20^ (SD_d_ = standard deviation of difference). The MDC_95_ was calculated using the equation MDC_95_ = 1.96 × SEM×√2,^8^ and the 95% LOA was calculated using the equation 95% LOA = mean difference ± 1.96 × SD_d_.^2^ A fixed bias was considered when zero did not lie within the 95% confidence interval for the mean difference of the two paired measures. On the other hand, proportional bias was considered when the regression between the difference and mean of the two paired measures was significant. The MDC_95_ is an indicator of the reproducibility and can be used to define the smallest amount of change needed to ascertain the occurrence of a real change beyond a measurement error when there is no systematic error (fix bias or proportional bias). The 95% LOA can be used to define the range in which repeated measurement might be expected to vary with the 95% confidence interval when there is a systematic error. The 95% LOA equation mentioned previously was used for fixed bias alone; on the other hand, for proportional bias, the variable of difference between two paired measures was replaced with its ratio to the mean of those measures.

## Results

This study included 60 healthy subjects with a mean age of 28.1 ± 11.6 years (range, 20-59 years). Among them, 36 were men, and 24 were women; in addition, 51 were right-handed and 9 were left-handed. They had a mean height of 165.2 ± 8.5 cm (150-180 cm), a mean weight of 58.3 ± 11.0 kg (36-99 kg), and a mean body mass index of 21.25 ± 3.09 kg/m^2^ (15.0-33.5 kg./m^2^).

**Figure 2:**
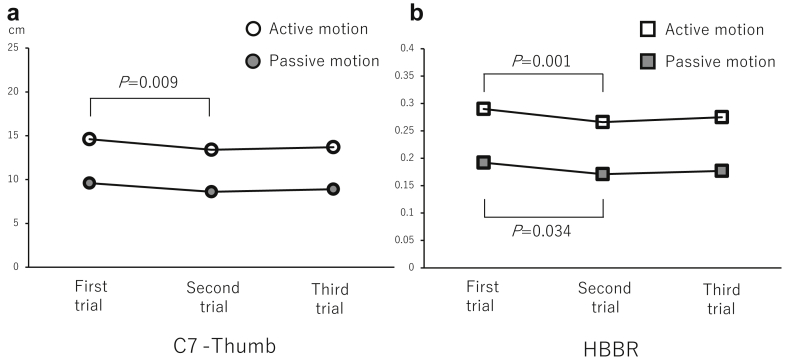
Changes of the C7-thumb (**a**) and the HBBR (**b**) in the active and the passive motion. *HBBR*, hand-behind-back ratio.

The mean time between the first and second trials was 4.0 ± 2.6 minutes (1-14 minutes) and that between the second and third trials was 1.5 ± 1.0 days (1-4 days). The descriptive measures of each trial are shown in Table I. The one-way repeated measure ANOVA revealed significant differences between the three trials regarding the active C7-thumb (F_2/118_ = 4.870; *P* = .009), active HBBR (F_2/118_ = 6.433; *P* = .002), and passive HBBR (F_2/118_ = 3.545; *P* = .032); however, no significant difference was observed for the C7-PSIS (F_2/118_ = 0.272; *P* = .762) and passive C7-thumb (F_2/118_ = 2.872; *P* = .061). The Bonferroni correction validated the significance between the first and second trials (Fig. 2).

**Table I:** Results of descriptive statistics of measures for each trial.

Variable	First trial	Second trial	Third trial
C7-PSIS (cm)	50.0 ± 4.1 (39-57)	50.1 ± 4.3 (41-61)	49.8 ± 4.6 (41-59)
Active-C7-thumb (cm)	14.6 ± 6.2 (0-31)	13.4 ± 5.6 (1-28)	13.7 ± 5.9 (0-31)
Passive-C7-thumb (cm)	9.6 ± 5.6 (-2-25)	8.6 ± 5.5 (-4-24)	8.9 ± 6.2 (-4-24 )
Active-HBBR	0.290 ± 0.116 (0.000-0.633)	0.266 ± 0.103 (0.070-0.542)	0.275 ± 0.110 (0.000-0.633)
Passive-HBBR	0.192 ± 0.108 (-0.040-0.455)	0.171 ± 0.104 (-0.075-0.438)	0.177 ± 0.117 (-0.082-0.490)

*PSIS*, posterior superior iliac spine; *HBBR*, hand-behind-back ratio.

The relative reliability of the measurement is shown in Table II. In accordance with the results of ANOVA, the ICCs for the active C7-thumb and HBBR were calculated with respect to each combination of two raters, excluding the results of the second trial. The ICCs for the HBBR ranged from 0.73 to 0.89, indicating moderate or good reliability for active and good reliability for passive measurement.

**Table II:** Relative reliability of measurement.

Variable	C7-PSIS	Active-C7-thumb	Passive-C7-thumb	Active-HBBR	Passive-HBBR
Inter all raters (N = 60)					
ICC_2,1_	0.72	-	0.83	-	-
(95% CI )	(0.50-0.84)		(0.76-0.89)		
Rater A vs. rater B (N = 20)					
ICC_2,1_	0.74	0.76	0.88	0.73	0.89
(95% CI )	(0.44-0.89)	(0.30-0.91)	(0.73-0.95)	(0.32-0.90)	(0.74-0.95)
Rater B vs. rater C (N = 20)					
ICC_2,1_	0.87	0.9	0.86	0.88	0.84
(95% CI )	(0.71-0.95)	(0.77-0.96)	(0.18-0.96)	(0.73-0.95)	(0.17-0.96)
Rater C vs. rater A (N = 20)					
ICC_2,1_	0.58	0.86	0.83	0.88	0.84
(95% CI )	(0.01-0.84)	(0.22-0.96)	(0.23-0.95)	(0.48-0.96)	(0.32-0.95)

*ICC*, intraclass correlation coefficient; *CI*, confidence interval; *PSIS*, posterior superior iliac spine; *HBBR*, hand-behind-back ratio.

In addition, the absolute reliability was analyzed excluding the measurements of the second trial (Table III). Bland-Altman analysis revealed that many, if not all, measurement items had fixed bias. Of those with no systematic errors, the MDC_95_ showed values of 0.053 and 0.036 for the active and passive HBBR, respectively.

**Table III:** Bland-Altman analysis for inter-rater reliability.

Variable	95% CI	Fixed bias	γ	*P* value of γ	Proportional bias	SEM	MDC_95_	95% LOA
Rater A vs. rater B (N = 20)								
C7-PSIS (cm)	−0.1 to 2.4	(-)	0.24	0.31	(-)	1.3	3.7	-
Active-C7-thumb (cm)	0.8 to 3.4	(+)	0.06	0.81	(-)	1.1	-	−3.2 to 7.4
Passive-C7-thumb (cm)	−0.4 to 1.6	(-)	0.33	0.16	(-)	0.7	1.9	-
Active-HBBR	0.011 to 0.059	(+)	0.17	0.48	(-)	0.023	-	−0.065 to 0.135
Passive-HBBR	−0.012 to 0.023	(-)	0.14	0.56	(-)	0.013	0.036	-
Rater B vs. rater C (N = 20) (-)								
C7-PSIS (cm)	−0.439 to 2.039	(-)	0.34	0.14	(-)	0.9	2.5	-
Active-C7-thumb (cm)	−0.7 to 2.1	(-)	0.44	0.05	(-)	0.8	2.3	-
Passive-C7-thumb (cm)	1.7 to 3.8	(+)	0.01	0.96	(-)	0.6	-	−1.8 to 7.3
Active-HBBR	−0.017 to 0.040	(-)	0.43	0.06	(-)	0.019	0.053	-
Passive-HBBR	0.032 to 0.076	(+)	0.02	0.93	(-)	0.013	-	−0.038 to 0.146
Rater C vs. rater A (N = 20) (-)								
C7-PSIS (cm)	−3.5 to −1.2	(+)	0.12	0.63	(-)	1.3	-	−7.2 to 2.5
Active-C7-thumb (cm)	−3.8 to −1.5	(+)	0.36	0.1	(-)	0.6	-	−7.3 to 2.0
Passive-C7-thumb (cm)	−4.0 to −1.5	(+)	0.04	0.88	(-)	0.8	-	−8.1 to 2.6
Active-HBBR	−0.065 to −0.019	(+)	0.24	0.3	(-)	0.013	-	−0.137 to 0.052
Passive-HBBR	−0.075 to −0.026	(+)	0.1	0.68	(-)	0.016	-	−0.155 to 0.054

*CI*, confidence interval; *SEM*, standard error of measurement; *MDC*_*95*_, 95% confidence interval of minimal detectable change; *LOA*, limit of agreement; *PSIS*, posterior superior iliac spine; *HBBR*, hand-behind-back ratio.

## Discussion

For estimating the vertebral body by palpation, the Jacoby and PSIS lines are used as references. The Jacoby line joins the superior aspect of the iliac crests and is commonly recognized to cross the L4-5 interspace or L4 spinous process. On the other hand, the PSIS line connects both PSISs, and its spinal level is known to be at the midpoint between the S1 and S2 foramen. However, there are individual variabilities in the course of these lines.^18^ In addition, identifying these lines by palpation does not always correspond to the true lines identified by X-ray.^12^ Furthermore, ascending/descending palpation along the spinal column is often difficult in subjects with obesity. These factors may affect the accuracy of identifying the spinal level using the HBB method.^9^ In this regard, reliability of the HBB method improves by limiting the palpation site to one location, using the distance between this site and the tip of the subject's thumb as a parameter of the range of internal rotation.^19^ This approach has been referred to as the measuring tape method.^10^ However, concern remains regarding the obstruction of the comparison between individuals of different physiques when using the measuring tape method. To address this concern, we normalized the tape measure to a ratio to a trunk length (reference trunk length).

For normalization, we considered the following landmarks as the reference trunk length: the C7-PSIS and distance between the point where the Jacoby line intersects the midline of the trunk and C7 spinous process (C7-Jacoby). A previous study^12^ found that identifying the PSIS line has better interobserver reproducibility than the Jacoby line. In addition, the longer length of the C7-PSIS compared with the C7-Jacoby may allow smaller variations in repeated measurements. To ensure the high reliability of measurement, we concluded that the C7-PSIS was more suitable for the reference trunk length than the C7-Jacoby.

In this study, Bland-Altman analysis indicated the presence of systematic errors. Fixed biases were observed in all three raters, and the influence of existence or nonexistence of clinical experience was inexplicit. Bland-Altman analysis is an approach for just a confined pair, and the results are difficult to generalize. Thus, it should be recognized that different results might be seen among the other raters. Meanwhile, the relative reliability of our measurement was shown to be moderate to good. This study confirmed that the numerical scale obtained by the proposed method was reliable for clinical use.

Numerous studies have shown the acute effect of joint stretching on the range of motion.^3^^,^^5^^,^^13^^,^^15^^,^^16^^,^^23^ More recently, Busch et al^5^ have reported the effect of static stretching on the shoulder joint in collegiate baseball players with the glenohumeral internal rotation deficit. Their intervention included the internal rotation stretch in 90° abducted position and the cross-body stretch; these have common action with the HBB method in that the posterior aspect of the capsule is primarily elongated. In their report, each stretch was held for 30 seconds, and immediately after the intervention, the range of internal rotation was increased with a prolonging effect of 60 minutes or more. The range of motion test may be considered to have the same action on the subject's body as the stretching exercise in that stretching stimuli are applied to the soft tissues around the joints. Holzgreve et al^11^ investigated the repetition-dependent acute effect of stretching on the range of motion in the application of the range of motion test. In their experiment, the subject performed 20 repetitions of passive horizontal abduction in which each repetition was held for about 3 seconds with an interval of 3 seconds. In consequence, range of motion revealed significant flexibility gains within 20 repetitions. We assumed that such an acute stretching effect might also occur in our experiment and that based on clinical experience, it would disappear after 1 day. For these reasons, we adopted the current experimental design in this study. As a result, ANOVA suggested the presence of acute stretching effect through significant changes in the C7-thumb and HBBR between the first and second trials. These findings indicate that when conducting the HBB method, immediate retest may target shoulders of different conditions; in addition, shoulders may be measured under similar conditions as the first trial by putting time one day.

This study has several limitations. First, the range of the subject's age was not wide enough. In particular, we had no participants under the age of 20 years. Because the concept of our method is to compare individuals of different physiques conveniently, it would be preferable to include children as study subjects. Second, only the inter-rater reliability was examined and not the intrarater reliability. Third, as with the conventional HBB method, those who cannot reach the spinal column or midline of the sacrum bone were not eligible for our procedure.

The HBBR may be used as a parameter of the shoulder internal rotation, which enables the comparison between individuals of different physiques. Further studies are warranted to validate the relationship of the HBBR with the internal rotation angle of the scapulohumeral joint and effect of body size disparity on the corresponding vertebral level.

## Conclusions

We proposed a modified measuring tape method as the HBB method. The tape measure was normalized to a ratio to a reference trunk length. The present study confirmed that the numerical scale obtained by this manner was reliable for clinical use. The measures by our procedure may be used as a parameter of the shoulder internal rotation which enables the comparison between individuals of different physiques.

## Disclaimers

Funding: No funding was disclosed by the authors.

Conflicts of interest: The authors, their immediate families, and any research foundation with which they are affiliated have not received any financial payments or other benefits from any commercial entity related to the subject of this article.
